# Asymmetrical Generalization of Length in the Rat

**DOI:** 10.1037/xan0000056

**Published:** 2015-04-27

**Authors:** Yutaka Kosaki, John M. Pearce

**Affiliations:** 1 School of Psychology, Cardiff University

**Keywords:** generalization of magnitude, spatial learning, discrimination of length

## Abstract

Two groups of rats in Experiment 1 were required to escape from a square pool by swimming to 1 of 2 submerged platforms that were situated beside the centers of 2 opposite walls. To help rats find a platform, black panels of equal width were pasted to the middle of the walls that were adjacent to the platforms. The width of the 2 panels was 50 cm for Group 50, and 100 cm for Group 100. Test trials were then conducted in the same pool, but with the platforms removed and with a 50-cm panel on 1 wall and a 100-cm panel on the opposite wall. Group 50 expressed a stronger preference for the 100-cm than the 50-cm panel during the test, whereas Group 100 expressed a similar preference for both panels. Thus the degree of generalization from the short to the long panel was greater than from the long to the short panel. Experiments 2 and 3 pointed to the same conclusion. They were of a similar design to Experiment 1, except that the lengths of the panels for the 2 groups were 25 and 50 cm in Experiment 2, and 25 and 100 cm in Experiment 3. The results are explained by assuming the original training results in the walls without black panels entering into inhibitory associations. This inhibition is then assumed to generalize more to the short than the long test panels and thereby result in an asymmetry in the gradients of generalization between the different lengths.

A characteristic of many accounts of stimulus generalization is the assumption that the gradient of generalization between two stimuli is symmetrical. Thus, for any given dimension, the extent of generalization from one stimulus to another will be the same, no matter which of them serves as the signal for reinforcement (e.g., Blough, 1975; Pearce, 1994; Spence, 1937). One justification for this assumption is that the amount of generalization between two stimuli is determined by their similarity (e.g., Shepard, 1987) and, since the similarity between two stimuli is often regarded as being symmetrical, it then follows that the extent of generalization between them will also be symmetrical. While this reasoning might hold for stimulus dimensions that are conventionally studied in conditioning experiments, such as the frequency of a tone, or the wavelength of a light, there is evidence to suggest that it does not hold for stimuli that differ in magnitude.

Scavio and Gormezano (1974), for example, conducted eyeblink conditioning with two groups of rabbits using a low intensity tone as the conditioned stimulus (CS) for one group, and a high intensity tone for the other. For the group trained with the loud tone, subsequent generalization tests with intensities lower than the training value revealed a conventional gradient, with responding becoming progressively weaker as the intensity of the tone was further removed from that used for training. In contrast, for the group trained with the weak tone, the strength of the conditioned response (CR) became progressively stronger as the distance between the intensity of the test and the training stimulus increased. Thus the degree of generalization from the weak to the strong stimulus was greater than from the strong to the weak stimulus. According to Razran (1949), a similar asymmetry was found in 67 experiments conducted in Pavlov’s laboratory between 1924 and 1941 using lights, whistles, and bells as the conditioned stimuli. The asymmetry has also been reported with humans, where the strength of the galvanic skin response to a tone that signaled shock was directly related to the intensity of the tone during generalization tests (Hovland, 1937). In contrast to conventional theories, therefore, it appears that when stimuli differ in magnitude, generalization from the weak to the strong one is considerably greater than from the strong to the weak one.

Kosaki, Jones, and Pearce (2013) referred to the foregoing asymmetry in order to explain their findings from a spatial learning experiment. Rats were required to escape from a square, gray pool by swimming to a submerged platform. A long black plastic panel was attached to each of one pair of opposing walls, and a short black plastic panel was attached to each of the two remaining walls. A platform was situated beside the middle of each long panel for one group, and the middle of each short panel for a second group. The group trained to swim to a platform beside the long, but not the short panels, readily solved its discrimination by acquiring a preference for searching near the long rather than the short panels. In contrast, the group trained with the platform beside the short panels found the discrimination much harder, so much so that even though the long panels were twice the length of the short panels, the discrimination was not mastered. In order to explain these results, Kosaki et al. (2013) suggested they were a consequence of an asymmetry in generalization based on stimulus magnitude.^1^ They proposed that a change in the length of an object might be equivalent to a change in its magnitude. If this were the case, then for the group trained with the platform beside the short panels, there would be considerable generalization from the short to the long panels, which would then make the discrimination hard to solve. The opposite of this effect in the group trained with the platform near the long panels would, in contrast, lead to the discrimination being relatively easy to solve.

Two obvious questions are raised by the foregoing discussion, both of which are considered in the present article. The first question is why should it be that stimulus generalization based on magnitude, including length, is asymmetrical? There are at least three possible answers to this question. The first to be considered is based on Hull’s (1949) concept of stimulus intensity dynamism, where the strength of a CR to a CS is assumed to be determined, in part, by the intensity of the stimulus that elicits it. If it is accepted that a wide object is more intense than a narrow one, then it would follow that training with a narrow panel, and testing with a wide panel will result in a stronger response to the test stimulus than when the wide panel is used for training and the narrow one is used for testing (see Grice & Saltz, 1950).

A rather different explanation for the asymmetry observed by Kosaki et al. (2013) stems back to the work of Spence (1937). This explanation was originally developed with experiments involving stimuli that differ in intensity (Mackintosh, 1974; see also Logan, 1954; Perkins, 1953), but it can be applied just as well when stimuli differ in length. If conditioning takes place with a stimulus of given length, then it will enter into an excitatory association. In addition, a representation of the absence of this stimulus, S_O_, by virtue of signaling the absence of reinforcement, will enter into an inhibitory association. Should a test trial be conducted with a smaller stimulus than the one used for training, then the test stimulus can be assumed to be more similar to S_O_ than the training stimulus, but if the test stimulus is larger than the training stimulus then it can be assumed to be less similar to S_O_ than the training stimulus. Given these differences, there will be more scope for the generalization of inhibition from S_O_ to the small than the large test stimulus, and thus the former will elicit a weaker conditioned response than the latter and result in generalization gradients that are asymmetrical.

The third explanation can be developed from proposals by Bouton and Hendrix (2011), which were used to explain the outcome of discriminations involving stimuli of different durations. It is possible to regard a stimulus of certain width as being composed of two elements, A and B, each of which may gain excitatory strength if the stimulus is paired with reward. If a test is then given with a shorter stimulus, which might excite A by itself, the removal of B will result in a weaker response than to the training stimulus. Conversely, a test with a longer stimulus than the one used for training might be construed as ABC. Since this transformation allows the continued presence of A and B, it follows from certain theories (e.g., Rescorla & Wagner, 1972) that the strength of the response to ABC will be similar to AB. In other words, there will be a generalization decrement when testing takes place with a stimulus that is shorter than the training stimulus, but not when the test stimulus is longer.

The second question to be considered in the present article is whether it is reasonable to assume that there is an asymmetry in stimulus generalization as far as the length of an object is concerned. To the best of our knowledge, only one study has investigated stimulus generalization when the length or, in this case, diameter of an object has been involved. Grice and Saltz (1950) trained rats to approach a white circle in order to gain food. Subsequent tests in which the rats were required to approach circles of different sizes revealed a more profound generalization decrement when the test circles were smaller than the circle used for training than when they were larger than the training circle. Indeed, when the diameter of the training circle was increased by just over 25%, there was no indication of any weakening of the response. The proposals of Kosaki et al. (2013) are consistent with this pattern of results, but they would be more convincing if stimulus generalization based on the length of objects was investigated with similar apparatus and stimuli to those used in the original studies by Kosaki et al. The three reported experiments were conducted with this goal in mind. As well as providing a test of the proposal by Kosaki et al. that generalization gradients based on the dimension of length are asymmetrical, the experiments may also permit an evaluation of the three different explanations that have just been outlined for this asymmetry.

## Experiment 1

In one of the experiments described by Kosaki et al. (2013), the width of the black plastic panels on one pair of opposing walls was 100 cm, while on the other pair of walls it was 50 cm. As noted above, the discrimination when the platform could be found in front of the 100-cm panels, but not the 50-cm panels, was acquired more readily than with the opposite arrangement. The implication of this finding, according to Kosaki et al., is that excitation generalized strongly from the 50-cm panels to the 100-cm panels, and relatively weakly from the 100-cm to the 50-cm panels. The purpose of the first experiment was to test this prediction.

**Figure 1 fig1:**
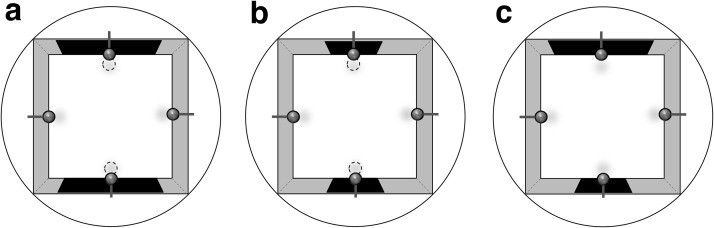
Schematic representations of the arenas used for training and testing in Experiment 1. The square arena set inside the circular swimming pool was used for Group 100 (Panel a) and Group 50 (Panel b). The two platforms, represented by dashed circles, were beside the black panels. Four landmarks, represented by gray circles, were attached to the middle of walls. Panel c shows the square arena used for the generalization test. A 100-cm panel and a 50-cm panel were pasted on two opposite walls. The generalization test trials were conducted in the absence of platforms.

Two groups of rats were first trained to escape from a square swimming pool by finding one of two escape platforms (see Figure 1). The platforms were situated close to the middle of two opposing walls to which were attached black plastic panels with a width of 100 cm for Group 100, and 50 cm for Group 50. There were no panels attached to the two remaining walls. The training phase was followed by a sequence of repeated test trials for which both platforms were removed from the pool, and one of the black panels was 50 cm wide and the other was 100 cm wide. Both groups were therefore given a choice between a familiar training panel and a novel test panel. If stimulus generalization based on the dimension of length is asymmetrical then, for the test with Group 100, rather little excitation should generalize from the large training panel to the small test panel, and considerably more time should be spent searching near the large rather than the small panel. On the other hand, for Group 50, considerable excitation should generalize to the large test panel from the small training one, and the preference for the training over the test panel should be smaller than for Group 100.[Fig-anchor fig1]

In keeping with the experiments by Kosaki et al. (2013), a landmark was attached to the top of each of the four walls, at the center. The purpose of this object was to facilitate the identification of the middle of the walls, and thus help rats find a platform. The landmarks were identical and, since they were attached to each wall, they could not be used to identify the walls that were adjacent to the platforms. Such identification could be achieved only by looking for a wall with a black panel.

### Method

#### Subjects

The subjects were 24 experimentally naïve, male, hooded-Lister rats supplied by Harlan Olac (Bicester, Oxon, U.K.). They were approximately 3-months old at the start of the experiment. All rats were housed in pairs in a temperature-controlled environment (approximately 20 °C) on a 12-h light–dark cycle with lights on at 0700. Rats had free access to food and water throughout the experiment.

#### Apparatus

The experiment was conducted in a white circular pool that was 2 m in diameter and 60 cm deep. The pool was filled to a depth of 30 cm with a mixture of water and white opacifier (500 ml, OP303B, supplied by Rohm and Haas, U.K.). This opaque mixture was maintained at a temperature of 25 °C (± 2 °C) and was changed daily. A white circular ceiling with a diameter of 2 m was suspended 1 m above the top edge of the pool, and was fitted with eight 45-W recessed spotlights. Each light was 22.5 cm in diameter. The lights were spaced evenly in a circle with diameter 1 m, concentric with the pool. In the center of the ceiling was a 30-cm hole into which a wide-angle video camera was fitted. Images from the camera were relayed to a monitor in an adjacent room, together with recording equipment, and a PC with tracking software (Watermaze Software, Edinburgh, U.K.). This software could be used to record each rat’s swim path, and to measure the amount of time spent in different areas of the pool. Four gray polyurethane boards were inserted into the pool to create the square-shaped arena. They were 141 cm in length, 60 cm high, and 4 mm thick. A panel of black plastic adhesive film (Deco d-c-fix) with a height of 45 cm and cut to lengths of either 50 cm or 100 cm was pasted onto and covered the middle of the two walls that were opposing each other. The two remaining walls were uncovered. The center of each panel was superimposed on a notional vertical line passing through the center of the wall (see Figure 1). The bottom of the black panels extended below the water surface, and the top of the panel was 2 cm below the top edge of the wall. A gray curtain was drawn around the pool throughout the experiment to exclude any extramaze cues. It was hung at a distance of 25 cm beyond the edge of the pool, and covered the entire height from the ceiling to below the pool’s edge.

Two identical, circular, clear-Perspex platforms with a diameter of 10 cm were placed into the pool. Each platform was mounted on a column so that its upper surface was 2 cm below the surface of the water. Each platform was positioned with its center 15 cm from the midpoint of one of the two walls with a black panel, on a notional line that was perpendicular to the wall.

Four identical balls, 10 cm in diameter and covered in colored cartoon characters, were used as landmarks. They were supported by Perspex horizontal rods attached to the middle of the top of each wall. The centers of the landmarks were positioned 15 cm away from the wall to which they were attached. When a landmark was above a platform, its center was directly above the center of the platform.

#### Procedure

At the start of the experiment, the rats were randomly assigned into two groups of equal number. For both groups, two escape platforms were situated beside the two black panels pasted onto two opposing walls of the square arena. For Group 100, the two black panels were 100-cm long. For Group 50, they were 50-cm long. The rats were trained for four trials per session, with an intertrial interval (ITI) of approximately 5 min. Each training trial started with a rat being released gently into the pool facing a corner. Rats were released from each of the four corners once in a session, in a randomly selected sequence. Once the rat found a platform it was allowed to remain on it for 20 sec before being picked up by the experimenter, dried with a towel, and returned to a carrying cage for the duration of the ITI. If the rat failed to find a platform in 60 sec, it was guided to the platform by the experimenter placing his finger just in front of the rat’s snout. During the trials, the experimenter remained in a small room adjacent to the testing room, where the pool could be observed on a monitor. In order to randomize any undesirable effect of cues outside the pool beyond the curtain, the arena was rotated by 90, 180, or 270 degrees in a random fashion from trial to trial.

Training was conducted in the above manner for the first eight sessions, which was followed by a preliminary test trial on the following day. For this test, the rat was released from the center of the arena, which was identical to that used for training, apart from the absence of the platforms, and allowed to swim for 60 sec. The purpose of this test was to determine if both groups had acquired a similar preference for searching in the regions where the platforms had been located, and in the equivalent regions adjacent to the walls without panels. The preliminary test was followed by two further sessions of training (Session 9 and 10). The experiment then concluded with a series of five test trials that were conducted on successive days. For each test trial, a black panel with a width of 100 cm was pasted to one wall, while a black panel with a width of 50 cm was pasted to the opposite wall (see Figure 1c). Other aspects of the apparatus were identical to those described for the first test trial. There were no panels attached to the remaining walls and the four landmarks remained attached to the walls. On each test trial the rats were released into the middle of the square arena and allowed to swim for 60 sec in the absence of the platforms. Across the five test trials, the rats were released into the middle of the pool facing one of the two walls without a black panel, in a randomized fashion. The square arena was also rotated randomly between test trials.

#### Behavioral measures and data analysis

Performance during the training trials was measured by recording the escape latency, which was the time taken for a rat to reach and climb onto a platform after being released into the pool. Performance during the test trials was measured by recording the time spent in circular zones (70-cm diameter) beside each wall, with the center of each zone coinciding with the center of the landmark attached to the top of the wall. For the preliminary test trial, the time spent in the two zones beside the two walls with a black panel, referred to as the “panel zones,” was compared with the time spent in the two zones beside the other two walls, referred to as “no panel zones.” For the generalization tests, the time spent in the zone beside the black panel of the same length that was used for training, referred to as the “training-panel zone,” was compared with the time spent in the zone by the panel of novel length on the opposite wall, referred to as the “test-panel zone.” Statistical analyses were conducted with a reliability of the effects assessed against a Type I error rate of .05 throughout the present report. If sphericity assumptions were violated in repeated measures analysis of variance (ANOVA), the Greenhouse-Geisser corrections were applied. The reported effect size for ANOVA with more than one factor is partial eta squared (η_p_^2^), while for comparisons between two means it is eta squared (η^2^). For both measures of effect size, 95% confidence intervals (CI) were computed using the method reported by Steiger (2004).

### Results

**Figure 2 fig2:**
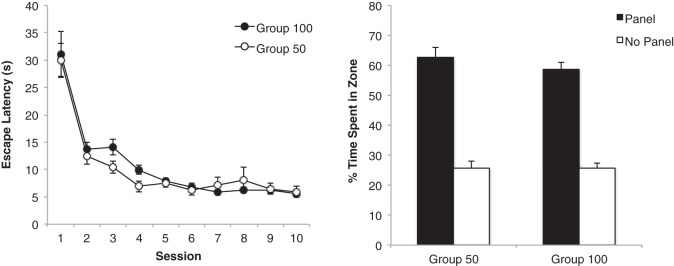
The mean escape latencies during the 10 sessions of training for the two groups of Experiment 1 (left-hand panel), and the mean percentages of time spent by the two groups of rats in the two zones in the first probe trial conducted between Session 8 and 9 (right-hand panel). The black bars represent time spent in two zones beside the two identical panels (panel zone). The white bars represent time spent in two zones beside the walls without panels (no panel zone). Error bars represent ± SEM.

The initial acquisition of the task by the two groups is shown in Figure 2. The two groups showed a similar reduction of escape latencies across 10 sessions of training. A group × session ANOVA revealed a significant main effect of session, *F*(9, 198) = 48.56, *p* < .001, η_p_^2^ = .69, 95% CI [0.60, 0.73], but the effect of group, and the interaction were not significant, *F*s < 1. The right-hand panel of Figure 2 shows the result from the preliminary test trial, which was conducted in the arena used for training. The two groups showed a similar preference for the panel zones over the no panel zones. A group × zone (panel vs. no panel) ANOVA revealed a significant effect of zone, *F*(1, 22) = 123.98, *p* < .001, η_p_^2^ = .85, 95% CI [0.69, 0.90], but the effect of group, *F*(1, 22) = 1.39, *p* > .1, and the interaction were not significant, *F* < 1.[Fig-anchor fig2]

**Figure 3 fig3:**
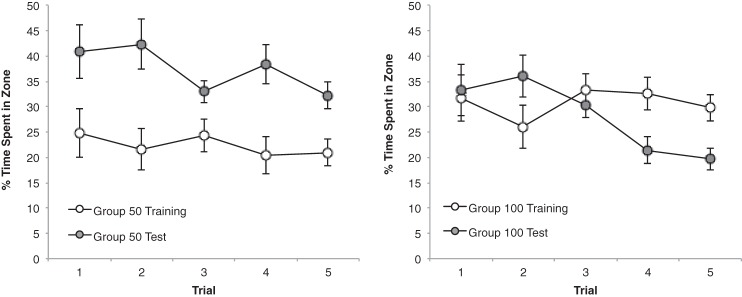
The mean percentages of time spent by Group 50 (left-hand panel) and Group 100 (right-hand panel) in the zone beside the training panel and the zone beside the test panel across five test trials of Experiment 1. Error bars represent ± SEM.

Our primary interest was the outcome of the generalization tests. The left-hand panel of Figure 3 shows that for each of the five test trials, Group 50 spent more time in the vicinity of the 100-cm test panel than the 50-cm training panel. From the right-hand panel, it is evident that Group 100 did not show a consistent preference for one panel length over the other. In support of this description, a Group × Zone × Trial ANOVA revealed a significant effect of zone, *F*(1, 22) = 4.73, *p* < .05, η_p_^2^ = .18, 95% CI [0.00, 0.43], and of trial, *F*(4, 88) = 11.44, *p* < .001, η_p_^2^ = .34, 95% CI [0.16, 0.45], and more importantly a Group × Zone interaction, *F*(1, 22) = 9.45, *p* < .01, η_p_^2^ = .30, 95% CI [0.03, 0.53]. The three-way interaction was not significant, *F* < 1. Tests of simple main effects, based on the Group × Zone interaction, confirmed that for the five test trials combined, Group 50 preferred the test panel over the training panel, *F*(1, 22) = 13.78, *p* < .005, η^2^ = .39, 95% CI [0.08, 0.59], but there was not a significant preference for one panel over the other in Group 100, *F* < 1 (the mean percentages of time spent by Group 100 in front of the training and test panels, respectively, was 30.7, and 28.1 for the five trials combined). In addition, Group 50 spent significantly more time in front of the test panel than did Group 100, *F*(1, 22) = 9.39, *p* < .01, η^2^ = .30, 95% CI [0.03, 0.53], and spent less time in front of the training panel than did Group 100, *F*(1, 22) = 6.85, *p* < .05, η^2^ = .24, 95% CI [0.01, 0.48].[Fig-anchor fig3]

The experiment revealed an asymmetry in stimulus generalization, but not quite in the manner predicted. It was suggested that during the test trials both groups would spend more time in front of the familiar training panel than the unfamiliar test panel, and that the extent of this preference would be greater for Group 100 than Group 50. In fact, for all the test trials combined, Group 100 spent a similar amount of time in front of each panel, which suggests there was considerable generalization from the 100-cm to the 50-cm panel. Furthermore, Group 50 spent substantially more time near the 100-cm test panel than the familiar 50-cm panel used for training. Thus the degree of generalization from the long training panel to the short test panel in Group 100 may have been considerable, but it was exceeded by the extent of generalization from the short to the long panel in Group 50.

Although the details of the experimental findings were unexpected, they nonetheless support the argument by Kosaki et al. (2013). The results imply that a discrimination of the form 100 + 50− was easier to solve than of the form 50 + 100−, because there was considerably more generalization from the reinforced to the nonreinforced cue in the latter than in the former. The results also have important implications for the three theoretical accounts considered in the Introduction but, to avoid undue repetition, a discussion of these implications will be postponed until the results from the next two experiments have been described.

## Experiment 2

Experiment 1 has shown that after training with a 100-cm panel, the response to the 50-cm-wide test panel was of similar magnitude to that directed to the training panel. The results of the previous experiment imply, however, that it should be possible to produce a response to the 50-cm test panel that is stronger than to a training panel if training takes placed with a panel that is less than 50 cm in width. In order to test this prediction, Experiment 2 was based on the same design as Experiment 1, except that the lengths of the panels were 25 and 50 cm, rather than 50 and 100 cm. Should the results be similar to those of Experiment 1, then more time will be spent near the 50-cm test panel than the 25-cm training panel in Group 25, whereas a similar amount of time will be spent near the two panels in Group 50. Such a pattern of results would strengthen the conclusion that there is an asymmetry in stimulus generalization based on the length of objects, by demonstrating both the reliability and generality of this effect.

### Method

#### Subjects

The subjects were 24 experimentally naïve male hooded Lister rats acquired from the same stock, housed in the same way, and of similar age to those used in Experiment 1.

#### Apparatus and procedure

The experiment was conducted in the same apparatus and with the same procedure as for Experiment 1, except that the widths of the panels for the training sessions were 50 cm for Group 50 and 25 cm for Group 25. The widths of the two panels that were present for the test sessions were 25 cm and 50 cm for both groups.

### Results

**Figure 4 fig4:**
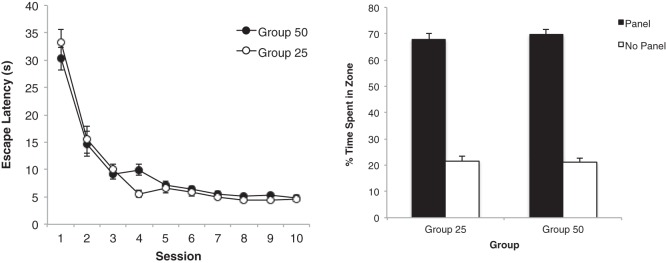
The mean escape latencies during the 10 sessions of training for the two groups of Experiment 2 (left-hand panel), and the mean percentages of time spent by the two groups in the two zones beside the black panels (panel) and two zones beside the walls without panels (no panel) during the first test trial. Error bars represent ± SEM.

The progressive reduction in the group mean escape latencies for each of the 10 sessions of training can be seen in the left-hand panel of Figure 4. Throughout this stage there was very little difference in the performance of the two groups. A Group × Session ANOVA revealed a significant main effect of session, *F*(9, 198) = 104.83, *p* < .001, η_p_^2^ = .83, 95% CI [0.78, 0.85], but the effect of group, *F* < 1, and the Group × Session interaction, *F*(9, 198) = 1.24, *p* > .1, were not significant.[Fig-anchor fig4]

The results from the preliminary test that took place in the arena that was used for training are displayed in the right-hand panel of Figure 4. Both groups spent considerably more time in the search zones in front of the two black panels than in the zones beside the walls without panels, and there was no difference between the groups. In support of these observations, a Group × Zone ANOVA revealed a significant effect of zone, *F*(1, 22) = 302.99, *p* < .001, η_p_^2^ = .93, 95% CI [0.86, 0.96], but no effect of group and the interaction was not significant, *F*s < 1.

**Figure 5 fig5:**
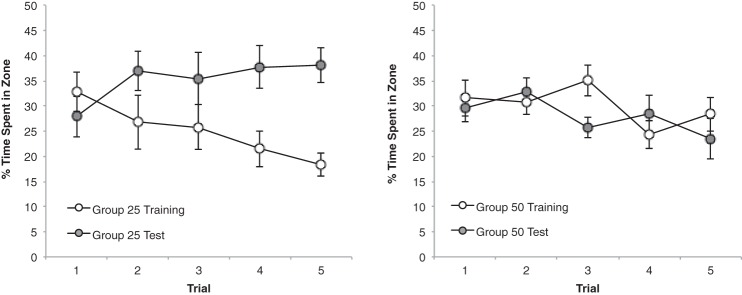
The mean percentages of time spent by Group 25 (left-hand panel) and Group 50 (right-hand panel) in the zone beside the training panel and the zone beside the test panel across five test trials of Experiment 2. Error bars represent ± SEM.

The results from the five test trials are shown in Figure 5. The pattern of results is strikingly similar to that obtained in Experiment 1. From the left-hand panel it is evident that Group 25 spent more time in the vicinity of the long test panel than the short training panel, particularly as testing progressed. By contrast, the right-hand panel shows that Group 50 spent a similar amount of time in front of both test panels. A Group × Zone × Trial ANOVA confirmed this description by revealing a significant Group × Zone interaction, *F*(1, 22) = 8.99, *p* < .01, η_p_^2^ = .29, 95% CI [0.03, 0.52]. There was also a significant effect of trial, *F*(4, 88) = 5.85, *p* < .001, η_p_^2^ = .21, 95% CI [0.05, 0.32], but the main effect of zone did not reach statistical significance, *F*(1, 22) = 4.03, *p* = .057. The three-way interaction was not significant, *F*(4, 88) = 1.16, *p* > .1. Subsequent analysis of simple main effects for the significant Group × Zone interaction, based on individual percentages of time for the five trials combined, revealed more time was spent by Group 25 beside the 50-cm than the 25-cm panel, *F*(1, 22) = 12.53, *p* < .005, η^2^ = .36, 95% CI [0.06, 0.58], whereas Group 50 spent a similar amount of time in front of both panels, *F* <1 (the mean percentages of time spent by Group 50 near the training and the test panels, respectively, was 30.0 and 28.0 for the five trials combined). The tests of simple main effects also revealed that significantly more time was spent near the test panel by Group 25 than Group 50, *F*(1, 22) = 8.98, *p* < .01, η^2^ = .29, 95% CI [0.03, 0.52], and significantly more time was spent near the training panel by Group 50 than Group 25, *F*(1, 22) = 4.63, *p* < .05, η^2^ = .17, 95% CI [0.00, 0.42].[Fig-anchor fig5]

Despite the use of panels that were 25 and 50 cm wide, rather than 50 and 100 cm wide, the results from the experiment were remarkably similar to those from Experiment 1. Thus the response to the 25-cm panels was much the same as to the 50-cm panels for Group 50, but there was a clear indication of a stronger response to the 50-cm than the 25-cm panels in Group 25. When comparing across the first two experiments, therefore, it is evident that the introduction of a 50-cm panel for testing resulted in a stronger response after training with panels that were shorter (25 cm) than the test panels than after training with longer (100 cm) panels.

Inspection of the left-hand panel of Figure 5 reveals that the difference between the amounts of time spent near the training and the test panels became more pronounced as testing progressed. It is hard to offer an explanation for this effect with confidence. However, it is possible that differences in the associative properties of the two panels were masked by a performance ceiling during the initial test trials, but that repeated nonreinforced exposure to the two panels permitted these differences to become more evident as the number of test trials increased.

## Experiment 3

The experiments thus far have shown there is an asymmetry in stimulus generalization based on length, when the lengths of the objects used for training and testing differ by a factor of two. The purpose of the final experiment was to determine if this asymmetry also exists when there is a fourfold difference between the widths of the panels used for training and testing. There were two reasons for conducting the experiment. First, Kosaki et al. (2013) presented rats with a discrimination involving panels that were either 25 cm wide, or 100 cm wide. In keeping with their other studies they found that the discrimination in which reward was signaled by the longer panel was acquired more readily than when it was signaled by the shorter panel. If this asymmetry in discrimination learning is based upon an asymmetry in stimulus generalization, then despite the large difference between the sizes of the panels, generalization from the small to the large one will be greater than from the large to the small one.

The second reason concerns the puzzling finding from the first two experiments, where there was no difference between the strength of the response during the test with the short and long panels after training with the long panels. In both experiments, the smaller width of the test than the training panels would be expected to result in a weaker response to the test than the training panels through a generalization decrement. One possible explanation for the failure to confirm this prediction is that the difference between the two panels was not sufficiently large. If this explanation is correct, then by increasing to a factor of four the difference between the widths of the training and test panels, it may now be possible to observe during testing a weaker response to the 25-cm than the 100-cm panels in the group trained with 100-cm-wide panels.

### Method

#### Subjects

The subjects were 28 experimentally naïve male hooded Lister rats acquired from the same stock, housed in the same way, and of similar age to those used in previous experiments.

#### Apparatus and procedure

The apparatus was the same as for the previous experiments, except that the lengths of the panels were 100 cm and 25 cm. Group 100 was trained with two, 100-cm panels and Group 25 was trained with two, 25-cm panels. Both groups received test trials with one 100-cm panel and one 25-cm panel. The remaining procedural details were the same as for Experiment 1.

### Results

**Figure 6 fig6:**
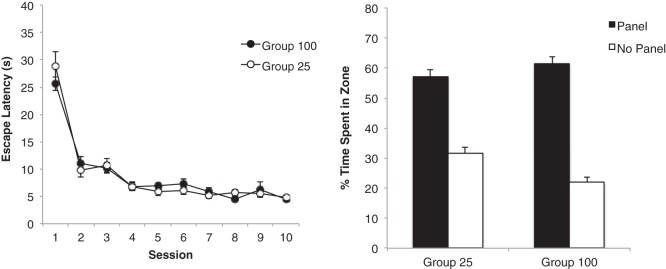
The mean escape latencies during the 10 sessions of training for the two groups of Experiment 3 (left-hand panel), and the mean percentages of time spent by the two groups of rats in the two zones beside the black panels (panel) and two zones beside the walls without panels (no panel) during the first test trial. Error bars represent ± SEM.

The group mean escape latencies for each of the 10 sessions of training are shown in the left-hand panel of Figure 6. As for the previous experiments, there was no difference between the two groups. A two-way ANOVA revealed a significant main effect of session, *F*(9, 234) = 91.43, *p* < .001, η_p_^2^ = .78, 95% CI [0.72, 0.81], but the main effect of group and the interaction were not significant, *F*s < 1.[Fig-anchor fig6]

The result from the preliminary test trial, which took place in the presence of the black panels that were used for training, can be seen in the right-hand panel of Figure 6. Although both groups spent considerably more time in the search zones beside the black panels than in the zones adjacent to the walls without a panel, the extent of this preference was greater for Group 100 than Group 25. In support of this description, a two-way ANOVA revealed a significant Group × Zone interaction, *F*(1, 26) = 6.61, *p* < .05, η_p_^2^ = .20, 95% CI [0.01, 0.43]. The effect of zone, *F*(1, 26) = 134.88, *p* < .001, η_p_^2^ = .84, 95% CI [0.69, 0.89], and group, *F*(1, 26) = 5.68, *p* < .05, η_p_^2^ = .18, 95% CI [0.00, 0.41], were also significant. Exploration of the interaction with tests of simple main effects revealed that both groups spent more time in the zones near the walls with, rather than without panels, *F*s(1,26) > 40.89, *p*s < .001, smallest η^2^ = .61, 95% CI [0.33, 0.74]. In addition, Group 25 spent more time near the walls without a panel than Group 100, *F*(1, 26) = 12.86, *p* < .01, η^2^ = .33, 95% CI [0.06, 0.54], but critically there was no difference between the groups in the amount of time spent near the walls with black panels, *F*(1, 26) = 1.89, *p* > .1

**Figure 7 fig7:**
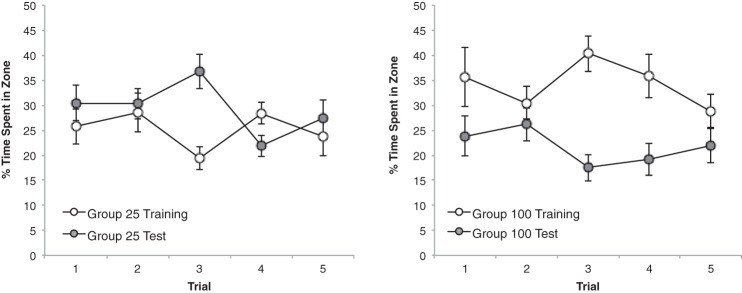
The mean percentages of time spent by Group 25 (left-hand panel) and Group 100 (right-hand panel) in the zone beside the training panel and the zone beside the test panel across five test trials of Experiment 3. Error bars represent ± SEM.

The results from the five test trials with the 25- and 100-cm panels are presented in Figure 7. From the left-hand panel it is evident that Group 25 spent approximately the same amount of time in the presence of both test panels over the five test trials whereas, the right-hand panel makes it clear that Group 100 expressed a stronger response for the training than the test panel.[Fig-anchor fig7]

A three-way ANOVA of individual results for the five test trials revealed a significant Group × Zone interaction, *F*(1, 26) = 18.81, *p* < .001, η_p_^2^ = .42, 95% CI [0.13, 0.61]. There was also a significant effect of trial, *F*(1, 26) = 4.72, *p* < .005, η_p_^2^ = .15, 95% CI [0.00, 0.39], zone, *F*(1, 26) = 4.66, *p* < .05, η_p_^2^ = .15, 95% CI [0.00, 0.38], but not group, *F* < 1. All interactions involving trial were not significant, *F*s(2.15, 55.92) < 2.07, *p*s > .1 A simple main effects analysis of the Group × Zone interaction revealed that Group 100 spent significantly more time near the 100-cm panel than the 25-cm panel, *F*(1, 26) = 21.10, *p* < .001, η^2^ = .45, 95% CI [0.15, 0.63], but the amount of time spent in each of these zones by Group 25 was not significantly different, *F*(1, 26) = 2.37, *p* > .1 (the mean percentage of time spent by Group 25 near the training and test panels, respectively, was 25.2 and 29.4 for the five trials combined). Additionally, it was found that Group 100 spent more time near the training panel than Group 25, *F*(1, 26) = 12.65, *p* < .005, η^2^ = .33, 95% CI [0.06, 0.54], and less time near the test panel than Group 25, *F*(1, 26) = 14.88, *p* < .005, η^2^ = .36, 95% CI [0.08, 0.57].

The experiment again demonstrated an asymmetry in generalization based on length. In keeping with the previous experiments, generalization from the familiar training panel to the unfamiliar test panel was greater when the training panel was short and the test panel was long (Group 25) than when the role of these two panels was reversed (Group 100). The pattern of this asymmetry was somewhat different to that observed in Experiments 1 and 2. In the present experiment, test trials with a stimulus that was considerably shorter than the one used for training, for Group 100, resulted in a significant reduction in the strength of conditioned responding, whereas in the previous experiments the equivalent test revealed no difference between the strength of the response to the training and test panels. Furthermore, when the length of the unfamiliar test panel was longer than of the familiar training panel, for Group 25, then, in contrast to the previous studies, there was only a hint, which was not statistically significant, of more time being spent near the test than the training panel. These contrasting outcomes are presumably a result of the greater difference between the lengths of the training and test stimuli in the present than in the previous experiments, which would reduce the effects of generalization in both groups.

## General Discussion

The experiments have demonstrated reliably an asymmetry in stimulus generalization based on the length of objects. After being trained to approach an object of a certain length, the tendency to approach an object of a different length was stronger when the test object was larger, rather than smaller, than the one used for training. Indeed, when the length of the test panel was twice the length of the training panel, then the strength of the response to the test panel was significantly stronger than to the one used for training.

**Figure 8 fig8:**
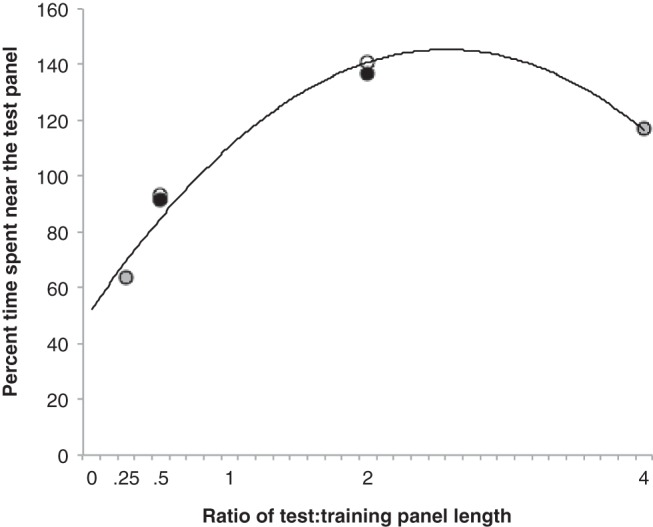
Generalization gradient of length based on the results from the three experiments. The length and the strength of responding across experimental conditions are normalized. Circles represent the actual data from the current experiments (black circles; Experiment 1, open circles; Experiment 2; gray circles; Experiment 3), and the curved line represents a fitted function.

In order to depict a generalization gradient based on all of the present findings, the results from each experiment were normalized by converting, for each subject, the mean percentage of time during all five test trials that was spent in the vicinity of the test panel, to a percentage of the mean time that was spent near the panel used for training. The results from the transformations can be seen in Figure 8, where the abscissa represents the ratio of the length of a test panel to the length of the training panel with which it was presented. It is evident from the figure that on those occasions when there were more than two results for the same ratio, the similarity between the results is remarkably close. Moreover, the figure makes quite clear that there is an asymmetry around the training: test ratio of one, in stimulus generalization based on the length of the panels.[Fig-anchor fig8]

The gradient in Figure 8 matches closely the pattern of results obtained by Grice and Saltz (1950), who conducted generalization tests with circles of different diameters after rats had been trained to approach a circle of a given diameter. When the test circles were smaller than the training circle, there was steady decline in the strength of responding as the difference between the training and test circles increased. In contrast, when the size of the test circles increased progressively from the training circle, there was initially no decrement in the strength of the response but a reduction in response strength was eventually observed. Contrary to the results from Experiments 1 and 2, Grice and Saltz did not observe a significantly stronger response to any test stimulus that was larger than the training stimulus, relative to the strength of response to the training stimulus. Despite these differences between the two experiments, which may be a consequence of the different methodologies that were used, when taken together the results from the two reports provide firm grounds for concluding there is an asymmetry in the stimulus generalization based on the length, or width, of an object.

With one exception, the present results are also in keeping with the results from experiments investigating generalization based on changes in stimulus intensity. From the results reported by Scavio and Gormezano (1974), and from results in Pavlov’s laboratory as described by Razran (1949), the strength of the conditioned response to a stimulus of any intensity, during a generalization test, appears to be a direct function of the intensity of the test stimulus. Thus, for any test stimulus that was weaker than the training stimulus, the response was weaker than to the training stimulus, whereas for any test stimulus that was stronger than the training stimulus the test response was stronger than to the training stimulus. The exception to this rule in the present experiment concerns the right-hand point shown in Figure 8. On the basis of the foregoing summary of findings, the value of this point should have been larger rather than smaller than the adjacent point to the left. Perhaps a similar pattern of results would have been reported by Scavio and Gormezano, and Pavlov, if their tests had included stimuli of greater intensity than the values employed. Alternatively, it is possible that generalization gradients based on the width of objects do not match exactly gradients based on intensities such as loudness.

The pattern of our results has important implications for the theoretical analysis of generalization gradients based on stimulus intensity. We shall consider first the idea that the intensity of a stimulus contributes to the strength of conditioned responding (Hull, 1949). It is conceivable that generalization gradients based on the width of an object are symmetrical, but the influence of stimulus intensity would be to enhance the strength of response to a test stimulus when it is wider, rather than narrower than the training stimulus. This explanation, of course, assumes that the width of a black panel determines its perceived intensity. In fact, there are several aspects of the present results that pose a challenge to the foregoing explanation. It follows directly from this account that during the training stage, the groups trained with the wider panels should have spent more time in front of these panels than the groups trained with the narrower panels. In contrast to this prediction, there was no indication during the preliminary test trial in each of the three experiments of more time being spent beside the training panel when it was wide rather than narrow. Furthermore, during the test trials in Experiment 3, Group 25 spent a similar amount of time in front of the 25-cm- and the 100-cm-wide panels. If the intensity of the panels directly influences the amount of time spent in front of them, then considerably more time should have been spent in front of the larger than the smaller panel.

Our results also provide scant support for the suggestion that the asymmetry in stimulus generalization based on length can be understood by assuming that a stimulus of large magnitude contains the same elements as a narrow stimulus, plus some additional elements (e.g., Bouton & Hendrix, 2011). According to this analysis, a narrow black panel might be construed as containing one element A, and a wide panel as containing two elements, AB. If AB serves as the cue for reward, then it follows from certain theories (e.g., Rescorla & Wagner, 1972) that when testing takes place with A, the response will be weaker than to AB. Conversely, after training with A, if AB is presented then the strength of the response to both stimuli will be similar because they both contain the critically important element, A, that was paired with reward. This analysis therefore predicts quite accurately the pattern of results observed in Experiment 3. Where the analysis falls down, however, is with explaining why the response to the 100-cm test panel in Group 50 of Experiment 1 was stronger than to the 50-cm training panel. If the 50-cm training panel is construed as being composed of one element, A, and the 100-cm test panel is construed of two elements, AB, then it is not at all easy to understand from this perspective how adding the notional element, B, resulted in a stronger response to the 100-cm rather than the 50-cm panel. A similar problem is posed by the results of Group 25 in Experiment 2.

We turn now to consider the possibility that the original training in each of the experiments resulted in a cue associated with the absence of a black panel, S_O_, entering into an inhibitory association (Mackintosh, 1974; Logan, 1954; Perkins, 1953). The present results are largely consistent with this proposal. If it is assumed that inhibition generalizes along the dimension of length from S_O_ to the different stimuli that were used for the test trials in each of the three experiments, then the extent of this generalization should be greater to test panels that are close to S_O_. For rats trained with a 50-cm-wide panel, it would then follow that a test trial with a 25-cm-wide panel will result in a relatively weak response because of considerable inhibition generalizing to it from S_O_, whereas the response to a 100-cm test panel should be relatively strong because the inhibition that generalizes to it from S_O_ will be slight. A comparison of the results in Experiments 1 and 2 is consistent with this prediction.

By appealing to the generalization of inhibition associated with S_O,_ it is also possible to account for the results in Experiments 1 and 2, where the response to a wide test panel was stronger than to a narrow training panel. Following from the proposals of Logan (1954) and Perkins (1953), Mackintosh (1974) has suggested that effects of this kind can be regarded as an instance of peak shift, where inhibition associated with S_O_ will shift the peak of responding to a test stimulus that is of greater magnitude than the training stimulus. To take this analysis a step further, according to the analysis of peak shift offered by Spence (1937), moving a test stimulus away from a reinforced stimulus in the direction that is also away from S_O_, will result in an increase in the strength of the response to the test stimulus, and then a decrease (see Purtle, 1973). The analysis thus explains why the response to a test stimulus was significantly stronger than to the training stimulus, when it was twice the size of the training stimulus (Experiments 1 and 2), but not when it was 4 times the size of the training stimulus (Experiment 3).

Despite these successes, there is an obvious cause for concern with an interpretation of our results in terms of the interaction between excitatory and inhibitory generalization gradients. At the heart of this interpretation is the assumption that the cues associated with the absence of the black panels, S_O_, entered into an inhibitory association. The present results are compatible with this assumption, but they do not provide any independent support for it. Further experiments are therefore needed to demonstrate that the training used in the above experiments results in S_O_ acquiring inhibitory associative strength. Until such a demonstration is forthcoming, this account for our findings should be regarded with a degree of caution.

The experiments confirm that generalization gradients based on the length of an object are asymmetrical. They also show for the first time that the generalization that takes place when there is an increase in the length of an object can result in a stronger response to a test stimulus than to the one used for training. None of the explanations we have considered is able to explain all of our findings, but perhaps the most satisfactory is the one that was considered last. Whatever the theoretical explanation for our findings, it seems reasonable to conclude that the asymmetry in the discrimination of length reported by Kosaki et al. (2013) was a consequence of the asymmetry in generalization based on this property of objects.

## Footnotes

1 Given that the panels were all of the same height, generalization might have been based on their area, rather than their length. In the absence of any evidence to choose between these alternatives, we shall continue to assume it was the length of the panels that was the relevant dimension. However, even if the area of the panels was the critical dimension, the argument put forward in the present article would remain the same.
